# Alterations in the renin-angiotensin system during septic shock

**DOI:** 10.1186/s13613-025-01463-x

**Published:** 2025-03-24

**Authors:** Camille Benaroua, Fabrizio Pucci, Marianne Rooman, Adrien Picod, Raphaël Favory, Matthieu Legrand, Jean-Louis Vincent, Jacques Creteur, Fabio Silvio Taccone, Filippo Annoni, Bruno Garcia

**Affiliations:** 1 Department of Intensive Care, Erasme Hospital, Hôpital Universitaire de Bruxelles, Université libre de Bruxelles (ULB), Brussels, Belgium; 2 Department of Intensive Care, Centre Hospitalier Universitaire de Lille, Lille, France; 3 BIO—Computational Biology and Bioinformatics, Université Libre de Bruxelles, Brussels, 1050 Belgium; 4 Université Paris Cité, UMR-S 942, INSERM, MASCOT, Paris, France; 5 Department of Anesthesia and Perioperative Care, Division of Critical Care Medicine, University of California, San Francisco, San Francisco, CA USA

**Keywords:** Sepsis, Distributive shock, Vasoplegia, Vasopressor, Angiotensin converting enzyme, Angiotensin II, Renin, Dipeptidyl peptidase 3

## Abstract

**Background:**

Alterations in the classical Renin-Angiotensin Aldosterone System (RAAS) have been described during septic shock and are associated with patient outcomes. Since the alternative RAAS has also been reported to be altered in critically ill patients, and given that the RAAS can be modulated by specific therapeutics, such as angiotensin II, understanding its pathophysiology is of primary interest.

**Objective:**

To describe the alterations in the classical and alternative RAAS during septic shock in comparison with healthy controls.

**Methods:**

This prospective, monocentric, controlled study enrolled 20 patients fulfilling the septic shock diagnosis, as defined by the Sepsis-3 criteria, along with 30 controls. The main exclusion criteria were the use of any prior medication modifying the RAAS, prior liver failure (Child-Pugh score > 9), or chronic kidney disease (estimated glomerular filtration rate < 30 ml/min/1.73 m²). Equilibrium concentrations of RAAS peptides were analyzed using a liquid chromatography-mass spectrometry method from heparinized plasma. Circulating angiotensin-converting enzyme (cACE), cACE type 2 (cACE2) activities, and circulating dipeptidyl peptidase 3 (cDPP3) concentrations were assessed. Values were measured at diagnosis, 6 h after diagnosis and on days 1 and 3. The main timepoint of interest was 6 h after diagnosis. Values 6 h after diagnosis were compared to 30 controls.

**Results:**

In septic shock patients, increased concentrations of the main peptides of the classical and alternative RAAS were observed compared to controls, particularly angiotensin I (Ang I) and angiotensin-(1–7) (Ang-(1–7)). Additionally, there was a significant increase in the Ang I/Ang II ratio (1.16 [0.74–3.31] vs. 0.34 [0.25–0.43], *p* < 0.05) and the Ang-(1–7)/Ang II ratio (0.15 [0.08–1.30] vs. 0.03 [0.02–0.04], *p* < 0.05). We also observed a significant reduction in cACE activity (3.38 [2.29–6.8] vs. 7.89 [6.39–9.05] nmol Ang II/L/h), an increase in cACE2 activity (814 [669–1987] vs. 214 [132–293] pmol Ang-(1–7)/L/h), and increased cDPP3 concentrations (54.6 [35–142.2] ng/mL vs. 13.7 [11.9–15.4] ng/mL, all *p* < 0.05).

**Conclusions:**

Septic shock was associated with increased Ang I/Ang II and Ang-(1–7)/Ang II ratios, along with reduced cACE activity, increased cACE2 activity, and elevated cDPP3 concentrations compared to healthy controls.

**Graphical abstract:**

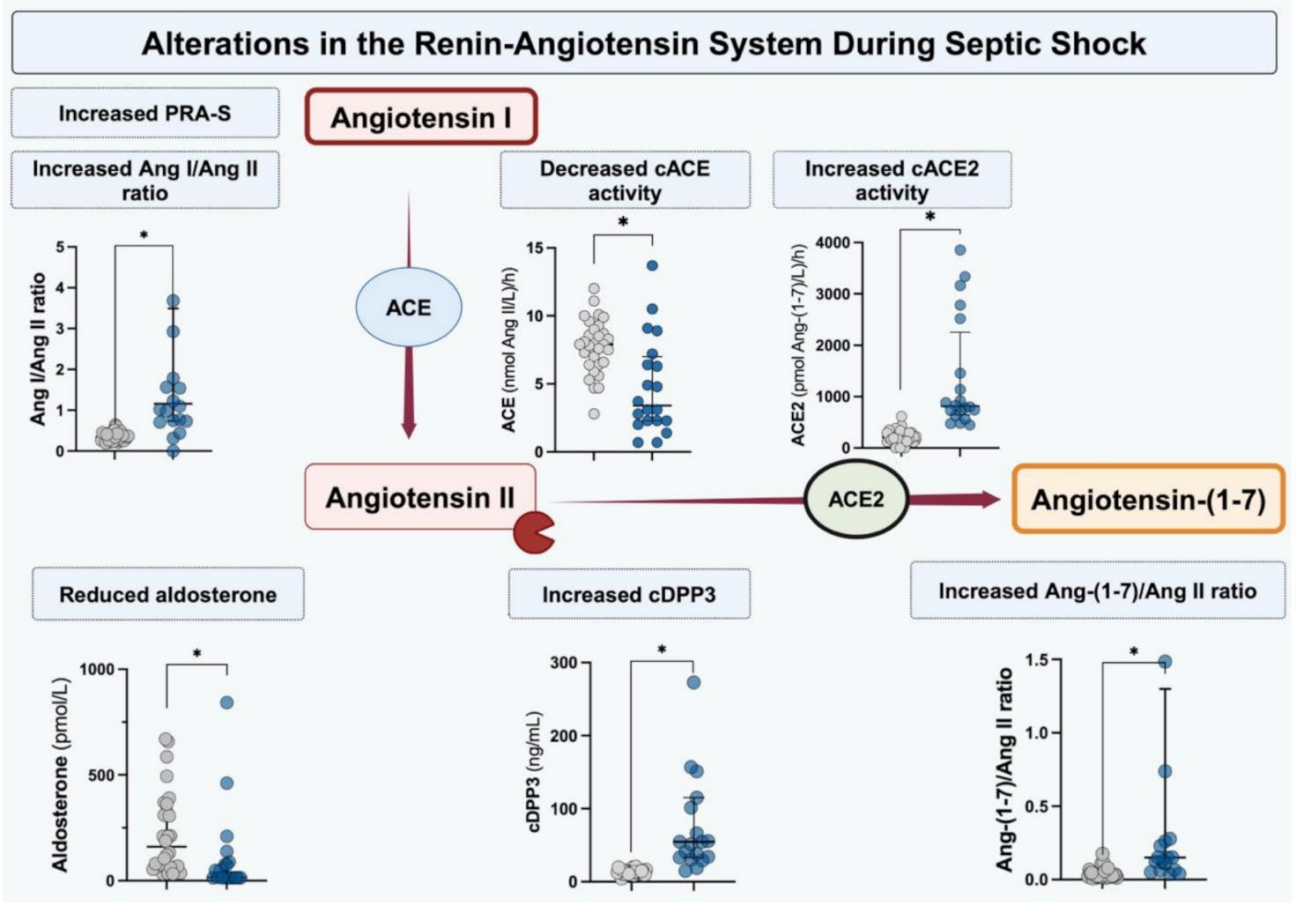

## Introduction

According to the Sepsis-3 Consensus for Sepsis and Septic Shock [1], sepsis is defined as life-threatening organ dysfunction caused by a dysregulated host response to infection, responsible for 11 million deaths worldwide [2]. Septic shock is characterized by circulatory failure and inadequate oxygen utilization at the cell level and is associated with high ICU mortality [3]. To counteract the circulatory failure observed during sepsis, hemodynamic management is based on the use of fluids and early norepinephrine administration [4]. In recent years, advances in understanding the pathophysiology of Renin Angiotensin Aldosterone System (RAAS) disturbances during sepsis and septic shock have led to increased interest in the use of alternative vasopressors, such as angiotensin II [4, 5]. However, despite the RAAS being a promising target for septic shock, supporting data remain limited [6, 7].

The classical RAAS is mediated by angiotensin II (Ang II), produced from the initial cleavage of angiotensinogen through the action of renin into angiotensin I (Ang I) [7], which is then converted into Ang II via angiotensin-converting enzyme (ACE). Recent research has also described another pathway, known as the “alternative” RAAS, which counterbalances the classical RAAS and is mediated through angiotensin-converting enzyme type 2 (ACE2) [8]. ACE2 converts Ang I and Ang II, respectively, into Angiotensin-(1–9) and Angiotensin-(1–7) (Ang-(1–7). Ang-(1–7) binds to a specific receptor (Mas), leading to a range of effects that counteract the ACE/Ang II/AT_1_ axis [9].

Increased renin concentrations have been observed in patients with catecholamine-resistant vasodilatory shock, correlating with a high Ang I/Ang II ratio, a marker associated with poor prognosis [10, 11]. Several pathophysiological hypotheses could explain these results, including reduced ACE activity, as shown in experimental sepsis or pediatric septic shock [12, 13] or increased degradation of Ang II, either by ACE2 or by peptidases, such as circulating dipeptidyl peptidase 3 (cDPP3) [11, 14, 15, 16, 17]. However, due to challenges associated with measuring the RAAS, descriptions of the various peptide concentrations and enzyme activities during septic shock are limited in the literature.

Our primary objective was, therefore, to describe the alterations of the classical and alternative RAAS during septic shock, along with circulating ACE and ACE2 activities and circulating DPP3 concentrations, in comparison to healthy controls. Additionally, we aimed at characterizing the evolution of RAAS alterations throughout the course of the disease.

## Methods

### Study design

This prospective study was conducted in the Department of Intensive Care at the Erasme hospital, Hôpital Universitaire de Bruxelles (HUB) from December 2021 to October 2023. The study was designed in accordance with legal and regulatory requirements, as well as the Guidelines for Good Clinical Practice and the Declaration of Helsinki. The protocol was approved by the local ethics committee (n°P2021/360/ B4062021000188). Patients or their representatives provided written informed consent for inclusion in the study and had the option to refuse participation.

### Study population

Patients were eligible for inclusion if they met the following criteria during screening: age > 18 years, septic shock as defined by the Sepsis-3 criteria within 6 h of diagnosis [18]. The exclusion criteria were as follows: an expected death within 48 h, personal medication with ACE inhibitors, angiotensin II receptor blockers or angiotensin receptor-neprilysin inhibitors, chronic kidney disease with an estimated glomerular filtration rate < 30 mL/min/1.73 m², chronic liver disease (e.g. Child-Pugh > 9), extracorporeal assistance and pregnancy. Healthy controls were selected from a pool of volunteers working in the ICU. Eligibility criteria were aligned with the exclusion criteria applied to septic shock patients.

After patient admission and obtaining written consent from the patient or their representatives, data regarding circulatory, pulmonary, renal, and hepatic functions were collected. Additionally, the Sepsis-related Organ Failure Assessment (SOFA) score and Acute Physiology and Chronic Health Evaluation II (APACHE II) score were calculated.

For angiotensin and enzymatic activity quantification, blood was collected in tubes containing lithium heparin and immediately centrifuged at 2000 x g for 15 min at 20 °C. Supernatant plasma was collected in pre-labeled aliquots and stored at -80 °C. Equilibrium angiotensin peptides were measured at *Attoquant diagnostics* (Vienna, Austria) using a liquid chromatography-tandem mass spectrometry method following 60 min of equilibration of conditioned lithium-heparin plasma at 37 °C and subsequent stabilization, as described previously [19]. The lower limit of quantification were as follows: <2 pmol/L for Angiotensin-(1–5) and Angiotensin IV, < 3 pmol/L for Ang-(1–7), < 2.5 pmol/L for Angiotensin III, and < 13.9 pmol/L for aldosterone. For enzymatic quantification, samples were spiked with their respective substrates (ang I for ACE and ang II for ACE2) and incubated at 37 °C with or without specific inhibitors (ACE: 10 µM lisinopril; ACE2: 10 µM ML N-4760) and liquid chromatography-tandem mass spectrometry was used to quantify the obtained products (angiotensin II from ACE activity; angiotensin-(1–7) from ACE2 activity) [20]. Angiotensin-based markers for renin (PRA-S) were derived from Ang II and Ang I levels by calculating their sum (PRA-S) as previously described [20]. Circulating DPP3 concentration was measured as previously described. The DPP3 assay is an immunoluminometric assay for the quantification of dipeptidyl peptidase 3 (DPP3) in human EDTA plasma. The luminescence signal (relative light units, RLU) is directly proportional to the DPP3 concentration in the plasma sample. Importantly, DPP3 concentration and activity are linearly and strictly correlated. The limit of detection (LoD) and limit of quantification (LoQ) for the DPP3 assay have been determined as 1.6 ng/mL. The assay shows no signal loss due to high DPP3 concentrations up to 2000 ng/mL (high-dose hook effect). The intra-assay and inter-assay coefficients of variation (CV) were 3.8% and 5.2%, respectively [21].

Four time points were considered for blood sampling: at septic shock diagnosis, 6 h after and on days 1 and 3. Values from the 6-hours time point were compared to those of 30 healthy controls.

### Study aims

The primary aim was to describe the equilibrium peptides of the RAAS, including Ang I, Ang II, Ang III, Ang IV, Ang-(1–7), and Ang-(1–5), as well as the circulating DPP3 concentration, and the cACE and cACE2 activities in comparison to healthy controls 6 h after the diagnosis of septic shock. Secondary aims included the evolution of the different components of the RAAS in relation to clinical and biological severity variables, including norepinephrine dose, lactate and SOFA score.

### Statistical analysis

Statistical analyses were conducted using IBM^®^ SPSS^®^ Statistics 27 for Windows, and figures were generated with Prism (GraphPad Software, Boston, MA). Continuous variables are presented as medians with interquartile ranges (IQR, 25th-75th percentiles), while categorical variables are expressed as counts and percentages. Group differences were assessed using the Mann-Whitney test. All tests were two-sided, with p-values less than 0.05 considered statistically significant.

## Results

### Demographic data

Out of 148 screened patients, 20 (13%) were included between December 2021 and October 2023, and 74 blood samples were analyzed at different time points. Patients were excluded due to the following criteria: refusal to participate (*n* = 44, 36%), use of medications interfering with the renin-angiotensin system (*n* = 41, 32%), norepinephrine already tapered or administered for more than 6 h (*n* = 19, 15%), chronic kidney disease (*n* = 12, 10%), chronic liver disease (*n* = 1, 1%), pregnancy (*n* = 1, 1%), absence of lactate measurement (*n* = 2, 1.6%), presence of extracorporeal circulatory assistance (*n* = 3, 2%), and death within the first 48 h (*n* = 5, 4%).

Patient characteristics are described in Table 1; 8 women (40%) and 12 men (60%) were included, with a median age of 69 [64–72] years. Most common comorbidities in patients with septic shock were chronic heart failure (25%), hypertension (9%), ischemic heart disease (25%), diabetes (35%), chronic kidney disease (15%), neoplastic diseases (45%), and immunodeficiency (40%). The primary sources of infection were the urinary tract (35%), respiratory system (30%), abdominal tract (30%), and soft tissue (5%). The median SOFA score at admission was 10 [8–10]. For the 12 out of 20 patients requiring invasive mechanical ventilation, the median ventilator-free days at 30 days (d) was 26d (IQR: 11–30). At 30d, 5 patients had died (25%), with 4 of these deaths occurring in the ICU. The median ICU length of stay was 5 [4–8] days.

**Table 1:** Demographic data at admissiondata are presented as counts and percentages or as medians with interquartile ranges

Variables	Septic Shock (*n* = 20)	Controls (*n* = 30)
Age, years [IQR]	69 [64–72]	43 [32–48]
Sex M, *N* (%)	12 (60%)	13 (43%)
BMI, kg/m^2^ [IQR]	23.9 [19.8–26.6]	23 [20.8–25.7]
Hypertension, *N* (%)	9 (45%)	1 (3%)
Chronic heart failure, *N* (%)	6 (30%)	0
Ischemic cardiomyopathy, *N* (%)	5 (25%)	0
COPD, *N* (%)	3 (15%)	0
Chronic respiratory disease, *N* (%)	2 (10%)	1 (3%)
Diabetes, *N* (%)	7 (35%)	0
Active cancer, *N* (%)	9 (45%)	1 (3%)
Chronic liver disease, *N* (%)	1 (5%)	0
Chronic kidney disease, *N* (%)	3 (15%)	0
Immunosuppression*, *N* (%)	8 (40%)	1 (3%)
APACHE II, points [IQR]	24 [19 -31]	-
SOFA, points [IQR]	10 [8 -11]	-
Sepsis type		-
Medical, *N* (%)	12 (60%)	
Surgical, *N* (%)	8 (40%)	
Primary source		-
Pulmonary system, *N* (%)	6 (30%)	
Urinary tract, *N* (%)	6 (30%)	
Abdomen tract, *N* (%)	6 (30%)	
Soft Tissue, *N* (%)	1 (5%)	
Organ Supports		-
AKI with dialysis/CRRT, *N* (%)	10 (50%)	
AKI with dialysis/CRRT, *N* (%)	4 (20%)	
Ventilator free days at 30 days [IQR]	26 [11–30]	-
ICU length of stay, days [IQR]	5 [4–8]	
ICU Mortality, *N* (%)	4 (20%)	
30-day Mortality, *N* (%)	5 (25%)	

**BMI** (Body Mass Index), **COPD**: Chronic Obstructive Pulmonary Disease; **APACHE II** (Acute Physiology and Chronic Health Evaluation II), **SOFA** (Sequential Organ Failure Assessment), **ICU** (Intensive Care Unit), **AKI** (Acute Kidney Injury), and **CRRT** (Continuous Renal Replacement Therapy)

*Immunosuppression was defined as the presence of any of the following: solid organ or bone marrow transplantation; prolonged corticosteroid therapy for more than 30 days or at a dosage exceeding 1 mg/kg/day of prednisone equivalent; immunosuppressive treatment; active solid cancer or cancer in remission for less than 5 years; neutropenia (absolute neutrophil count < 0.5 G/L) lasting more than 7 days; HIV infection; or genetic or acquired immunodeficiency

### Clinical and biologic parameter of patients

At 6 h after septic shock onset, hemodynamic data were as follows: heart rate of 102 [91–117] beats per minute, mean arterial pressure of 68 [60–76] mmHg, with an infusion rate of norepinephrine base at 0.68 [0.49–1.15] µg/kg*min. Median arterial lactate was 4 [2.9–6.3] mmol/L and the Glasgow Coma Scale score was 13 [3–15] (Table 2). The median urine output was 0.6 [0.4-1.0] mL/kg*h. Nine (45%) patients had acute kidney injury (AKI) as defined by the KDIGO classification, among whom 4 (20%) required renal replacement therapy (RRT). In terms of ventilatory support, 2 (10%) did not receive oxygen, 6 (30%) of patients needed high-flow nasal cannula, non-invasive positive pressure ventilation, or a venturi mask, while 12 (60%) were on invasive mechanical ventilation. Fifteen (75%) of patients received hydrocortisone (200 mg per day) and fludrocortisone (50 µg per day) supplementation when norepinephrine requirements exceeded 0.05 µg/kg/min.

**Table 2:** Characteristics of the septic shock patients 6 h after diagnosis compared to controlsdata are presented as counts and percentages or as medians with interquartile ranges

H6 after diagnosis	Septic Shock (*n* = 20)	Controls (*n* = 30)
Clinical variables		
MAP (mmHg)	68 [60–76]	71 [65–78]
Heart rate (/min)	102 [91–117]	93 [87–93]
CVP (mmHg)	8 [3–10]	-
Respiratory rate (/min)	24 [22–30]	22 [18–21]
Neurologic status Glasgow	13 [3–15]	15 [15–15]
Urine Output (ml/kg/h)	0.6 [0.4–1]	-
Temperature (°C)	37 [36.6–37.6]	36.2 [35.8–36.5]
Hemodynamic variables		
Norepinephrine (µg/kg/min)	0.68 [0.49–1.15]	-
Lactate	4.1 [2.9–6.3]	-
SvO_2_	75 [63–78]	-
Gap CO_2_	7 [6–10]	-
Ventilation variables		
ARDS (Berlin definition)		
Mild P/F 200–300	7	-
Moderate P/F 100/200	0	-
Severe P/F < 100	0	-
Non invasive ventilation / HFNC, *N* (%)	6 (30%)	-
Invasive mechanical ventilation, *N* (%)	12 (60%)	-
PaO_2_ /FiO_2_	358 [296–440]	-
Kidney variables		
Plasma creatinine (mg/dL)	1.68 [1.43–2.43]	-
Urinary creatinine (mg/dL)	56 [37.5–81]	-
AKI	9 (45%)	-
AKI with dialysis/CRRT, *N* (%)	4 (20%)	
Hepatic parameter		
Total Bilirubin, mg/dL (IQR)	0.74 [0.49–1.25]	-
Platelets (10^9^/L)	166 [54–256]	-
Inflammatory Marker		
Leucocytes (10^9^/L)	11.2 [3–23.4]	-
CRP (mg/L)	250 [125–390]	-

**AKI** - Acute Kidney Injury, **CRP** - C-Reactive Protein, **CVP** - Central Venous Pressure, **CO₂** - Carbon Dioxide (Gap CO₂), **HFNC** - High-Flow Nasal Cannula (Non-invasive ventilation or HFNC), **MAP** - Mean Arterial Pressure, **PaO₂/FiO₂** - Partial Pressure of Oxygen/Fraction of Inspired Oxygen, **RRT** - Renal Replacement Therapy, **SvO₂** - Mixed Venous Oxygen Saturation. Values are reported as median [interquartile range]

### RAAS in patients with septic shock and healthy controls

In the septic shock group, Ang I was significantly increased to 168 [33–1429] pmol/L compared to 36 [22–54] pmol/L in the control group (*p* < 0.05). In contrast, no significant differences were observed in plasma Ang II, with levels of 55 [20–229] pmol/L in the septic shock group vs. 114 [68–162] pmol/L in controls (*p* = 0.7). Additionally, notable increase in Ang-(1–7) was observed in the septic shock group: 15 [3–145] pmol/L compared to below detection < 3 [< 3–<3] pmol/L in the healthy control group (*p* < 0.05). No significant differences were observed in Ang III, Ang IV or Ang-(1–5) concentrations between groups (Table 3).

**Table 3:** Equilibrium RAAS profile, septic shock at H6 vs. healthy controls

	Septic Shock	Healthy controls
Ang I (pmol/L)	168 [33–1429] *	36 [22–54]
Ang II (pmol/L)	55 [20–229]	114 [68–162]
Ang-(1–7) (pmol/L)	15 [< 3–145] *	< 3 [< 3–<3]
Ang III (pmol/L)	< 2.5 [< 2.5–11]	5.1 [3.1–7.2]
Ang IV (pmol/L)	2.3 [< 2–13]	6.1 [4.4–9.1]
Ang-(1–5) (pmol/L)	8 [3.6–14]	5 [2.8–6.1]

**Ang I** - Angiotensin I (pmol/L); *p = 0.004 between septic shock and controls, **Ang II** - Angiotensin II (pmol/L), **Ang-(1–7)** - Angiotensin-(1–7) (pmol/L) *p < 0.001 between septic shock and controls

**Ang III** - Angiotensin III (pmol/L), **Ang IV** - Angiotensin IV (pmol/L), **Ang-(1–5)** - Angiotensin-(1–5) (pmol/L)

**Fig. 1:**
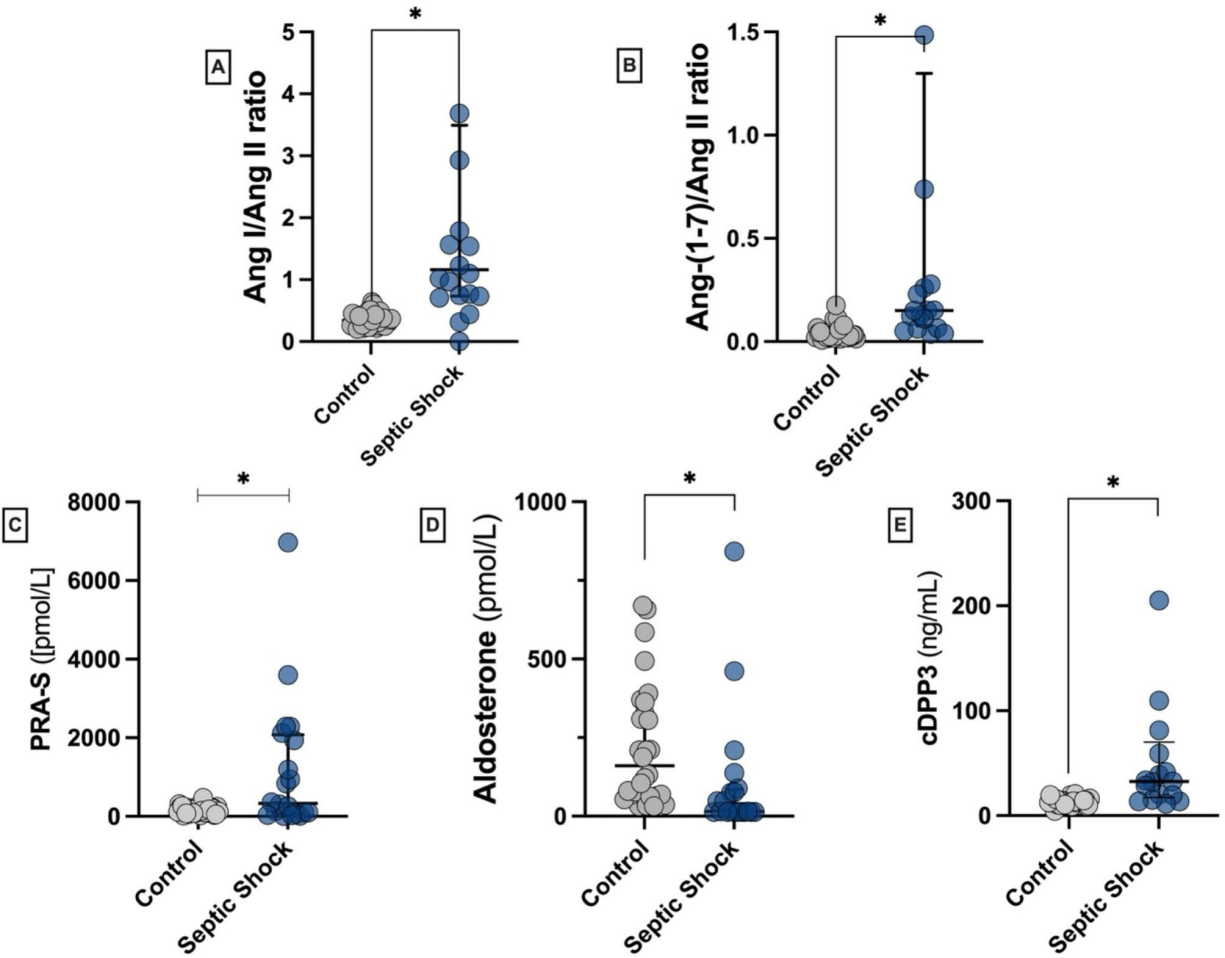
Angiotensin I/Angiotensin II ratio **(A)**, Angiotensin-(1–7)/Angiotensin II ratio **(B)**, Plasma Renin Activity-S **(C)**, Aldosterone **(D)** and cDPP3 **(E)** concentrations at 6 h after diagnosis compared to controls. *p < 0.05 between septic shock and control groups

The Ang I/Ang II ratio was significantly higher in the septic shock group compared to the control group (1.16 [0.74–3.31] vs. 0.34 [0.25–0.43], *p* < 0.001). Similarly, the Ang-(1–7)/Ang II ratio was higher in the septic shock group than the other (0.15 [0.08–1.30] vs. 0.03 [0.02–0.04] *p* < 0.001- Fig. 1).

Increased circulating DPP3 concentrations were observed in patients with septic shock (54.6 [35–142.2] ng/mL) compared to the control group (13.7 [11.9–15.4] ng/mL, *p* < 0.001). Furthermore, aldosterone concentrations were decreased in septic shock patients (15.5 [0–79.4] pmol/L) compared to the control group (160 [68.7–353.4] pmol/L, *p* < 0.001), Fig. 1.

**Fig. 2:**
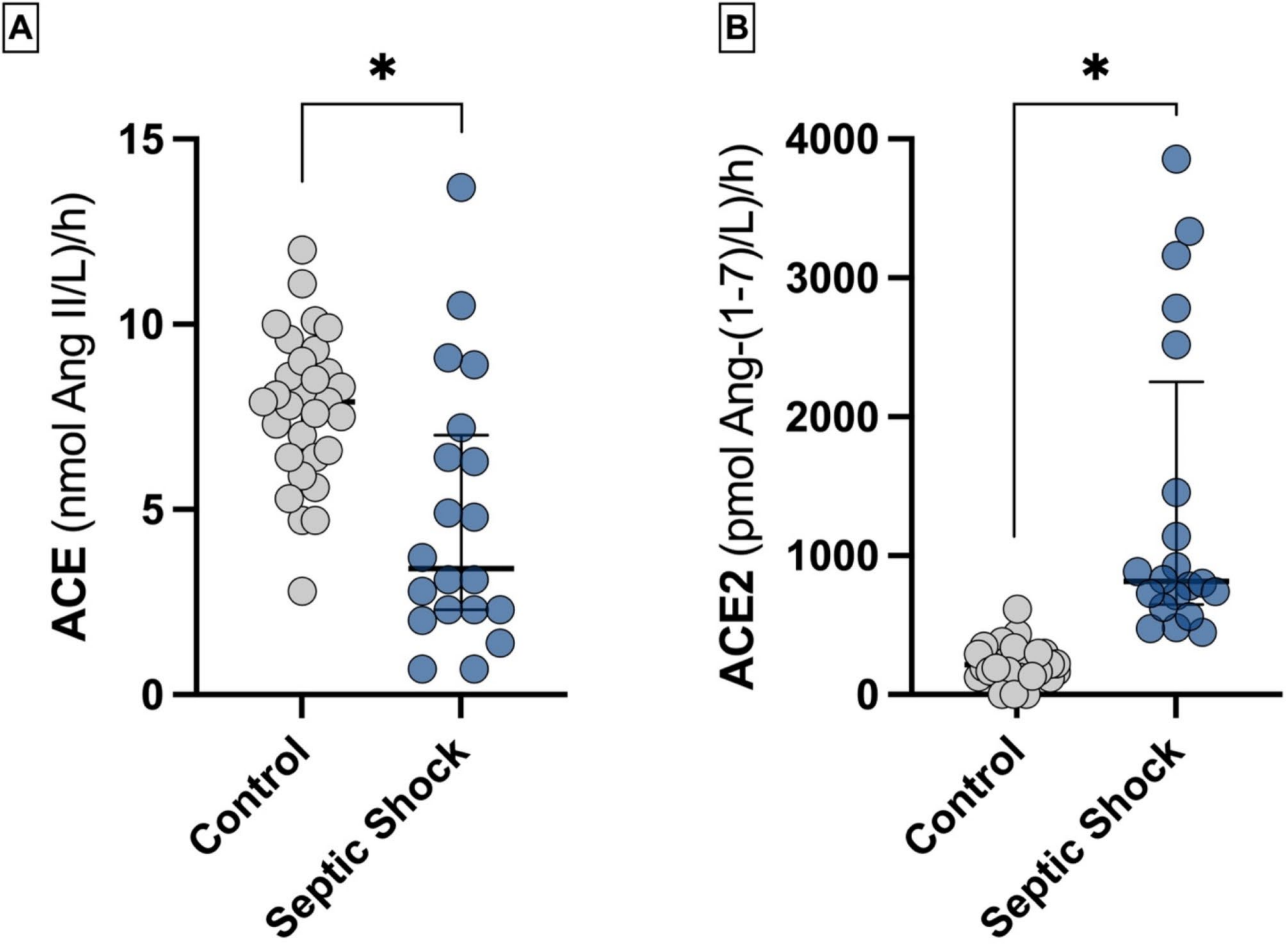
cACE and cACE2 activities 6 h after shock diagnosis. *p < 0.05 between septic shock and control groups

Patients with septic shock showed significantly lower cACE activity compared to controls (3.38 [2.29–6.8] vs. 7.89 [6.39–9.05] nmol Ang II production/L*h, *p* = 0.001). Conversely, cACE2 activity was markedly increased in septic shock patients (814 [669–1987] vs. 214 [132–293] pmol Ang 1–7 production/L*h - *p* < 0.001; Fig. 2).

### Renin angiotensin system evolution over time

The evolution of clinical and RAAS variables over time is described in Table 4 and 5. The differences observed at H6 were consistent across the study timeline, including an increased Ang I/Ang II ratio, an increased Ang-(1–7)/Ang II ratio, and elevated cDPP3 concentrations.

**Table 4:** Evolution of the clinical variables across the study timelinedata are presented as median [interquartile range]

	H0 (*n* = 20)	H6 (*n* = 20)	Day 1 (*n* = 18)	Day 3 (*n* = 16)	Controls (*n* = 30)
Clinical Data					
MAP (mmHg)	65 [63–73]	68 [60–75]	78 [71–83]	74 [73–82]	71 [65–78]
Heart rate (/min)	115 [98–124] *	102 [91 -117] *	99 [82–112]	91 [71–103]	93 [87–98]
Glasgow Coma Scale	14 [9–15]	13 [3–15]	14 [5–15]	14 [11–15]	15
Norepinephrine (µg/kg/min)	0.58 [0.42–0.69]	0.68 [0.5–1.12]	0.25 [0.17–0.57]	0.02 [0–0.01]	-
Biological Data					
Lactate (mmol/L)	4.3 [3.2–7]	4.05 [2.88–6.93]	2.8 [1.5–4.6]	1.6 [1.4–1.9]	-
SvO2 (%)	67 [61–81]	75 [63–78]	68 [64–74]	63 [60–68]	-
Creatinine (mg/dl)	1.96 [1.22–2.36]	1.68 [1.43–2.43]	1.72 [1.22–2.2]	0.99 [0.8–2.05]	-
CRP (mg/L)	190 [81–325]	250 [125–390]	270 [170–395]	210 [170–290]	-
APACHE score	23 [18–31]	24 [19–31]	22 [15–30]	19 [14–23]	-
SOFA Score	9 [8–11]	10 [8–11]	11 [9–12]	9 [8–11]	-

**MAP** - Mean Arterial Pressure (mmHg), **SvO₂** - Mixed Venous Oxygen Saturation (%), **CRP** - C-Reactive Protein (mg/L), **APACHE score** - Acute Physiology and Chronic Health Evaluation score, **SOFA Score** - Sequential Organ Failure Assessment Score. * *p*-value H0 < 0.001 H6 = 0.03 vs. healthy controls

**Table 5:** Evolution of the components of the RAAS across the study timeline data are presented as median [interquartile range]

	H0 (*n* = 20)	H6 (*n* = 20)	Day 1 (*n* = 18)	Day 3 (*n* = 16)	Controls (*n* = 30)
PRA-S (pmol/L)	515 [43–2561]*	333 [90-1985]*	171 [74–727]	51[26–128]*	143 [88–215]
Ang I (pmol/L)	221 [22–1618] *	168 [33–1428] *	83 [25–395] *	18 [7–79]	36 [22–54]
Ang II (pmol/L)	93 [18–376]	55 [20–229]	60 [17–114]	23 [13–35] *	114 [68–162]
Ang-(1–7) (pmol/L)	20 [< 3–142] *	15.3 [3.4–146] *	7.1 [< 3–34] *	< 3 [< 3–11] *	< 3 [< 3–<3]
Ang III (pmol/L)	< 2.5 [< 2.5–18]	< 2.5 [< 2.5–11]	< 2.5 [< 2.5–8]	< 2.5 [< 2.5–<2.5]*	5.1 [3.1–7.2]
Ang IV (pmol/L)	2.5 [< 2–20.8]	2.3 [< 2–13]	2.6 [< 2–7.4]	< 2 [< 2–2.3]*	6.1 [4.4–9.1]
Ang-(1–5) (pmol/L)	9.6 [2.4–23.8]	9 [4–15]	4.9 [2.8–15.8]	2.1 [< 2–8.1]	5 [2.8–6.1]
cDPP3 (ng/mL)	46.7 [32.8–103.9] *	54.6 [35–142.2] *	30.7 [18.4–51.6] *	20.1 [16.8–37.6] *	13.6 [11.7 -15.1]
Aldosterone (pmol/l)	48.4 [< 13.9–133.8] *	15.5 [< 13.9–79.4] *	< 13.9 [< 13.9–15] *	< 13.9 [< 13.9–51.2] *	160 [68.7–353.4]
Ang I/Ang II ratio	0.99 [0.62–1.68] *	1.16 [0.74–3.12] *	0.87 [0.67–1.28] *	0.62 [0.41–1.55] *	0.34 [0.26–0.42]
Ang-(1–7)/Ang II ratio	0.15 [0.08–1.4] *	0.15 [0.08–1.30]*	0.10 [0.07–0.39] *	0.23 [0.17–0.35] *	0.03 [0.02–0.04]
Aldosterone/PRA-S ratio	0.22 [0.02–0.52]*	0.12 [0.04–0.43]*	0.09 [0.03–0.19]*	0.59 [0.23–0.86]*	1.7 [0.4–2.4]
cACE activity nmol Ang II/L*h	4.41 [2.21–6.8] *	3.38 [2.29–6.8] *	4.48 [2.2–5.83] *	3.88 [2.73–5.62] *	7.89 [6.39–9.04]
cACE2 activity pmol Ang-(1–7)/L*h	910 [573–2314] *	814 [669–1987] *	807 [558–1781] *	1432 [585–3242] *	215 [132 ; 293]

**PRA-S –** Plasma Renin Activity *p value H0 = 0.03, H6 = 0.04, D1 = 0.64, D3 = 0.006, **Ang I** - Angiotensin I (pmol/L), *p value H0 = 0.008, H6 = 0.004, D1 = 0.1, D3 = 0.44 **Ang II** - Angiotensin II (pmol/L), p value H0 = 0.69, H6 = 0.25, D1 = 0.05, D3 < 0.001 **Ang-(1–7)** - Angiotensin-(1–7) (pmol/L), p value H0 < 0.001 H6 < 0.001 D1 < 0.001 D3 = 0.008; **Ang III** - Angiotensin III (pmol/L), * p value H0 = 0.55 H6 = 0.2 D1 = 0.07 D3 < 0.001 **Ang IV** - Angiotensin IV (pmol/L), p value H0 = 0.49 H6 = 0.1 D1 = 0.54 D3 = 0.29 **Ang-(1–5)** - Angiotensin-(1–5) (pmol/L), * p value H0 = 0.07 H6 = 0.07 D1 = 0.54 D3 = 0.29 **DPP 3** - Dipeptidyl Peptidase 3 (ng/mL), * p value H0 < 0.001 H6 < 0.001 D1 < 0.001 D3 < 0.001; Aldosterone pmol/l – * p value H0 = 0.007 H6 < 0.001 D1 < 0.001 D3 < 0.001 **Ang I/Ang II** - Ratio of Angiotensin I to Angiotensin II, p value H0 < 0.001 H6 < 0.001 D1 < 0.001 D3 = 0.001 **Ang-(1–7)/Ang II** - Ratio of Angiotensin-(1–7) to Angiotensin II, p value H0 < 0.001 H6 < 0.001 D1 < 0.001 D3 = 0.008. **Aldosterone/PRA-S ratio**: p value H0 < 0.001 H6 < 0.001 D1 < 0.001 D3 < 0.001; **cACE activity** - Circulating Angiotensin-Converting Enzyme activity (nmol Ang II/L/h), p value H0 < 0.001 H6 = 0.001 D1 < 0.001 D3 < 0.001. **cACE2 activity** - Circulating Angiotensin-Converting Enzyme 2 activity (pmol Ang-(1–7)/L/h. p value H0 < 0.001 H6 < 0.001 D1 < 0.001 D3 < 0.001

## Discussion

### Key findings

In the current study, using a high-quality and validated mass spectrometry method, we reported the primary peptides of the classical and alternative RAAS, along with the main enzyme activities during septic shock. The key findings are as follows: (1) we confirmed the previously described increase in the Ang I/Ang II ratio, reduced ACE activity, and decrease aldosterone concentration; (2) we observed a shift toward the activation of the alternative RAAS, indicated by an increased cACE2 activity and Ang-(1–7)/Ang II ratio; (3) these alterations may be driven by the reduction in cACE activity, DPP3 release, and/or by the increase in cACE2 activity.

### Interpretation of the data and implications of the study findings

The increase in the Ang I/Ang II ratio has already been described in a *post hoc* study of the ATHOS-3 trial, where, compared to controls, Ang I was elevated without a corresponding increase in Ang II, leading to a higher Ang I/Ang II ratio [10]. The authors hypothesized that this increase might be associated with reduced ACE activity in the context of endothelial dysfunction, which prevents the conversion of Ang I into Ang II [11]. This hypothesis is further supported by a recent pediatric study that found reduced ACE activity in septic shock [12]. Our findings align with this hypothesis, as we also observed reduced cACE activity, consistent with an experimental study in a sepsis swine model [13]. Several hypotheses have been proposed, ranging from endothelial dysfunction to the presence of circulating ACE inhibitors [11, 12]. However, the identity of a potential ACE inhibitor remains unclear, and as this study did not directly assess ACE protein concentration, further research is required to confirm its presence and significance [12]. The reduced cACE activity over the 3-day follow-up period, alongside the improvement in norepinephrine requirements and the Ang I/Ang II ratio, highlights that alterations in the Ang I/Ang II ratio are multifactorial and may be driven by DPP3 [16].

The increased renin activity with hypoaldosteronism observed in our study aligns with previous findings in septic shock, where this increase was associated with increased renal failure [22]. This observation, along with high Ang I concentrations, highlights the importance of the relative deficit in Ang II in the pathophysiology of septic shock. A decrease in the interaction between Ang II and AT_1_ might explain the reduced aldosterone concentrations observed.

This increase in the Ang I/II ratio might also be explained, in part, by other pathophysiological mechanisms related to the degradation of circulating Ang II by cDPP3 or cACE2 [6]. First, cDPP3 has been associated with worse outcomes in a prospective, observational international study involving 585 septic patients [17]. Since DPP3 is known to hydrolyze Ang II but not Ang I, the release of circulating DPP3 might contribute to the increased Ang I/Ang II ratio [23, 24]. An experimental study performed in swine using a monoclonal antibody able to neutralize circulating DPP3 supports this hypothesis. The neutralization of circulating DPP3 activity resulted in reduced norepinephrine and fluid requirements, along with higher Ang II concentrations, and a preserved Ang I/Ang II ratio [16]. Of note, in healthy humans and septic shock patients, there is a strict correlation between cDPP3 concentration and activity [17]. Despite the release of DPP3, no increase in Ang IV concentrations—metabolites of Ang II—was observed. This absence of increase could be attributed to DPP3’s specific effects on Ang IV as well, as evidenced by a mouse study where administration of DPP3 was associated with reduced Ang IV concentrations, highlighting the enzyme’s preference for smaller peptides [23].

The observed shift toward the alternative axis, with a higher Ang-(1–7)/Ang II ratio, suggests that the elevated Ang I/Ang II ratio might also be due to the enhanced degradation of Ang II into Ang-(1–7). This is supported by our finding of increased cACE2 activity compared to controls. The increase in cACE2 activity has already been suggested in the literature, with similar observations in the context of acute respiratory distress syndrome related to COVID-19. In those cases, both Ang-(1–7) levels and cACE2 activity were found to be elevated and associated with disease severity, a response hypothesized by the authors to be a response to injury [25]. Decreased ACE activity might also contribute to a reduction in Ang-(1–7) metabolism into Ang-(1–5), thereby further increasing Ang-(1–7) concentrations [9].

Another peptidase called neprilysin (NEP), can bypass ACE by directly generating angiotensin-(1–7) from angiotensin I. Several studies found a rise of circulating NEP concentration in critically ill, not correlated to prognosis [26].

A recent post hoc analysis of the Vitamin C, Thiamine, and Steroids in Sepsis (VICTAS) Trial utilized different methods for RAAS quantification, including ELISA for angiotensinogen measurement, radioimmunoassay for peptide levels, and fluorescence assays for enzyme activities [27]. In this study, the authors divided the cohort into a normal renin sepsis group and a high renin sepsis group, comparing both to a control group of healthy subjects. They reported reduced angiotensinogen levels compared to the control and normal sepsis groups, no differences in angiotensin II concentrations between the high renin group and controls, while angiotensin-(1–7) and DPP3 levels were increased. ACE activity was reduced, while ACE2 activity was increased. Our study, using a mass spectrometry method in a selected population of septic shock, confirms the alterations in the RAAS described in this study, with a decrease in the classical RAAS and an increase in the alternative RAAS. Additionally, the reduced angiotensinogen concentration, which has been reported to have a stronger association with mortality compared to lactate and renin, might contribute to the defect in Ang II concentrations observed in our study [28].

These findings suggest that the pathophysiology of RAAS alterations during septic shock is multifactorial, involving ACE dysfunction, increased ACE2 activity, and DPP3 release into the circulation. Targeting the RAAS during septic shock might aim to restore the Ang I/Ang II ratio, potentially through the use of ang II as a vasopressor and/or the development of drugs targeting circulating DPP3, such as procizumab. However, it remains unclear which patients would benefit from these strategies, and future approaches to phenotyping septic shock patients, such as using renin concentration to identify those who may have improved outcomes with angiotensin II, will be required [11].

### Strengths and limitations

This study is the first to describe both the classical and alternative RAAS pathways, including cACE and cACE2 activities, in a population of septic shock patients without any medications or medical conditions other than septic shock that could affect the RAAS. These analyses were conducted using a high-quality mass spectrometry method in a specialized laboratory. Our findings confirm various hypotheses proposed in recent years in the literature and provide significant insights into the pathophysiology of the RAAS during septic shock [6, 7, 9, 29]. However, our study has some limitations. The first major limitation is the small sample size, suggesting that our results are exploratory and should be confirmed by a large multicenter study. The methods for measuring RAAS metabolites also have certain limitations. Specifically, we assessed the equilibrium RAAS, which reflects a balance based on the respective activities of all circulating enzymes and peptide concentrations within the sample. However, the equilibrium measurement might overlook the potential role of membrane-associated enzyme activity and receptor-mediated clearance. Additionally, our assessment of angiotensin-converting enzymes activities was limited to the circulating moieties of these enzymes, a membrane-bound form, mainly expressed by pulmonary endothelial cells, and a soluble or circulating forms. The total enzyme activity therefore, remains unknown. Only enzymatic activity was assessed, while enzyme concentrations were not measured. Additionally, the study compared the values 6 h after diagnosis to those of the control group. This time point was chosen as it reflects early alterations of the RAAS following the resuscitation phase with fluids, which is the period during which interventions targeting the RAAS might be relevant [5]. The use of healthy volunteers as the control group in our study may have amplified the observed RAAS alterations during septic shock, as they do not share the same baseline comorbidities, chronic medications, or underlying pathologies typically seen in critically ill patients. This mismatch may have contributed to an overestimation of the differences in RAAS biomarkers. The absence of medications and/or conditions that can affect the RAAS might have resulted in a selected population that is not representative of those typically admitted to the ICU. Specifically, a high Ang I/Ang II ratio has been logically associated with the use of ACE inhibitors [11]. Future studies incorporating a hospitalized control group with comparable comorbidities, medication use, and pathologies but without septic shock could provide a more accurate and clinically relevant comparison. The absence of angiotensin-(1–9) and angiotensin-(1–12) quantification represents another limitation, as their inclusion could have provided a more comprehensive understanding of the full spectrum of RAS alterations observed in septic shock. Another limitation is the absence of measurements of NEP activity. However, during septic shock, a previous study suggested that NEP activity is low due to competitive substrates, explaining the lack of association between NEP concentration and outcomes [30]. The assessment of previous kidney function, including the absence of medical records to identify potential underlying kidney disease in some patients, represents a limitation of this study. The final limitation is the predominance of urinary sources of sepsis. It is noteworthy that such sources are not typically associated with severe refractory septic shock and high mortality [31].

## Conclusions

Septic shock was associated with an increased Ang I/Ang II ratio and a shift toward the alternative RAAS, as evidenced by an increased Ang-(1–7)/Ang II ratio. This shift might be explained by a decrease in circulating ACE activity, increased circulating ACE2 activity, and/or DPP3 release. While these alterations suggest potential therapeutic strategies, such as angiotensin II use or DPP3 inhibition, future research should determine whether these interventions can effectively reverse RAAS imbalances and improve outcomes.

## Data Availability

The datasets used during the current study are available from the corresponding author on reasonable request.
